# Effect of a Multifunctional Biosurfactant Extract Obtained from Corn Steep Liquor on Orange and Apple Juices

**DOI:** 10.3390/foods11213506

**Published:** 2022-11-04

**Authors:** Benita Pérez-Cid, Lorena Rodríguez-López, Ana Belén Moldes, José Manuel Cruz, Xanel Vecino

**Affiliations:** 1 Chemical Engineering Department, School of Industrial Engineering—Research Center in Technologies, Energy and Industrial Processes (CINTECX), University of Vigo, Campus As Lagoas-Marcosende, 36310 Vigo, Spain; 2 Food and Analytical Chemistry Department, Faculty of Chemistry, University of Vigo, Campus As Lagoas-Marcosende, 36310 Vigo, Spain

**Keywords:** fruit juices, biosurfactant extract, storage time, pH, biomass, sugars

## Abstract

Biosurfactant extracts are multifunctional ingredients composed of natural polymers that can be used in the food industry as stabilizing and antimicrobial agents, although their inclusion in food matrices has been scarcely explored. In this work, a biosurfactant extract, with antimicrobial properties, obtained from a fermented stream of the corn wet-milling industry was introduced into an apple and orange juice matrix to evaluate the changes produced in the sugar consumption, pH, and biomass formation at different temperatures (4–36 °C) and storage time (1–7 days). It was observed that the addition of biosurfactant extract reduced the hydrolysis rate of polymeric sugars, decreasing the concentration of soluble sugars from 85.4 g/L to 49.0 g/L in apple juice after 7 days at 20 °C in the absence and presence of biosurfactant extract, respectively. In general, soluble sugars increased in juices for 5–6 days and after those sugars decreased at different rates depending on the temperature of storage. Differences in sugar solubilization and degradation were more significant in apple juice than in orange juice at 20 °C and 7 days of storage, achieving for orange juice values of 101 and 102 g/L in the absence and presence of biosurfactant extract, respectively. Biomass growth was almost unaffected by the biosurfactant extract concentration and the optimal conditions for biomass production were detected at intermediated temperatures after 6–7 days of storage for both apple and orange juices, obtaining maximum concentrations of 1.68 g/L and 1.54 g/L for apple juice and orange juice, respectively, in the absence of biosurfactant extract. The pH during storage was kept in the range of 3.35–3.48 for apple juice and of 3.40–3.77 for orange juice.

## 1. Introduction

Biosurfactants are natural compounds of microbial origin, composed of a combination of lipids, sugars, or peptides, the most studied biosurfactants being the lipopeptides produced by *Bacillus subtilis*, the rhamnolipids produced by *Pseudomona aeruginosa,* and the sophorolipids produced by *Starmerella bombicola*. Biosurfactants can be very useful in different industrial sectors, including the food industry, to obtain more biocompatible, biodegradable, and stable formulations [1]. In fact, between 2021 and 2022, 115 reviews were published in the Scopus databse about the production, uses, and applications of biosurfactants, demonstrating the current interest of these surface-active compounds, the application of biosurfactants in the food industry being one of the less-studied fields. It is well-known that biosurfactants present emulsifying, stabilizing, and detergency capacities [2,3,4,5]. Additionally, some authors have demonstrated that biosurfactants possess antimicrobial and antiadhesive activities; however, despite these properties, there are few studies about the direct application of biosurfactants in food formulations or food matrices. Additionally, biosurfactants can be used as emulsifiers and thickeners since they can improve the texture, stability, volume, and preservation of baked foods [6]. For example, Hoffmann et al. [7] compared several physical parameters such as the interfacial tension, surface tension, and critical micellar concentration of different surfactants, including lipopeptide surfactin, rhamnolipids, sophorolipids gum arabic, lecithin, Tween 20, Tween 80, and polysorbates, among others, observing that surfactin does not form strong viscoelastic interfaces in emulsions but possesses a high interfacial charge, producing stable emulsions between pH 6 and 9 and NaCl concentrations up to 2.9%, improving the results obtained with lecithin. These characteristics are important for the use of biosurfactants as promising ingredients in food emulsion formulations. Ribeiro et al. [8] used a biosurfactant produced by *Saccharomyces cerevisiae* on salad dressing formulations. This biosurfactant was obtained in a controlled fermentation using a mixture of soybean, waste frying oil, and corn steep liquor as a carbon source and nutrients. The isolated biosurfactant was added to the dressing formulations at different concentrations (0.2–0.8%) together with Tween 80 and xanthan. These authors found that the biosurfactant in the salad dressings had a satisfactory pH and stability in comparison with the control formulation, in the absence of biosurfactant, and the rheological tests demonstrated the potential of the biosurfactant to act as a thickener in the presence of commercial emulsifiers. Other works suggest that biosurfactants promote the growth of probiotic bacteria such as *Lactobacillus casei* or *Lactobacillus delbrueckii*. Thus, López-Prieto et al. [9] evaluated the effects of the biosurfactant extract, obtained from corn steep liquor (CSL), which contains lipopeptides among other bioactive compounds, in a drinkable yogurt, observing that it increased the microbial biomass of *Lactobacillus casei*. The same effect was observed by Ravindran et al. [10], but using other lipopeptide, in this case produced by *Bacillus licheniformis*. This biosurfactant enhanced the survival of *Streptomyces thermophilus* and *Lactobacillus delbrueckii subsp*. *bulgaricus* and improved the stability, texture, and shelf life of yogurts. In another work, Martínez-Arcos et al. [11] used the biosurfactant extract obtained from CSL as coating agent on grapes, observing that it increased the self-life of grapes and reduced their roughness. Recently, Scalzini et al. [12] evaluated its effect as a stabilizing and solubilizing agent in the maceration of red wine, concluding that the biosurfactant extract obtained from CSL favors the extraction of anthocyanins from the skins during the maceration stage, increasing the antioxidant capacity of red wine. However, no studies currently exist about the effect of biosurfactants in juice matrices.

The aim of this work was to study the effect of a multifunctional biosurfactant extract obtained from CSL, composed of lipopeptides and antioxidants, in the sugar consumption, pH, and biomass production of juice. Two different juices were evaluated, natural orange juice and pasteurized apple juice, and different independent variables were selected: concentration of biosurfactant extract, storage time, and temperature.

## 2. Materials and Methods

### 2.1. Obtention of a Multifunctional Biosurfactant Extract from Corn Steep Liquor

The biosurfactant extract was obtained from CSL by performing a liquid–liquid extraction with ethyl acetate as the organic solvent. The extraction process conditions were as follows: CSL:ethyl acetate ratio was 1:3 (*v*/*v*) at 25 °C during an extraction time of 45 min with a shaker system at 150 rpm. Once the extraction was carried out, the organic solvent was evaporated by vacuum distillation (rotavapor R-120; Büchi Labortechnik, Flawil, Switzerland), and then the extract was redissolved in an aqueous solution following the experimental procedure described in a previous work [13]. In addition, CSL was provided by the Companhia Portuguesa de Amidos, S.A. (COPAM) (San João da Talha, Portugal).

### 2.2. Factorial Design to Evaluate the Effect of a Biosurfactant Extract in Orange and Apple Juices

Two different juice samples, natural orange juice and pasteurized apple juice, were subjected to a Box–Behnken factorial design [14]. The independent variables used in the study were the biosurfactant concentration (x_1_), storage time (x_2_), and temperature (x_3_), whereas the dependent variables were biomass concentration (y_1_), sugar concentration (y_2_), and pH (y_3_) for both juices. Table 1 summarizes the independent and dependent variables as well as the equations to code any values of the independent variables in the range of study.

Before the factorial design, natural orange juice from the Mercadona supermarket (Hacienda Fresh brand, Spain) was centrifuged at 4863 rcf, for 30 min at 4 °C; to discard the orange pulp, it was then vacuum filtered using a filter paper (60 g/cm^2^ weight).

The 15 experiments that constitute the experimental matrix of Box–Behnken factorial design (Table 2) were carried out by adding 25 mL of a solution of natural orange juice or pasteurized apple juice to a 50 mL Falcon tube, in the presence of different concentrations of biosurfactant extract (0–1 g/L); temperature (4–36 °C); and storage time (1–7 days). All experiments were performed in duplicate, although it is important to note that trials 13, 14, and 15 of the Box–Behnken factorial design represent the central point of the statistical model, in which all independent variables reach their central values, to stablish the reproducibility of the studied system. At the established time, juices were centrifuged at 4863 rcf, for 30 min at 4 °C, to separate the liquid and solid phases. The solid phase containing the biomass was washed twice with ultrapure water and its initial volume of water (25 mL) was restored for subsequent measurement by turbidimetry. In addition, an aliquot of the obtained supernatant (5 mL) was filtered through a 0.45 µm polytetrafluoroethylene (PTFE) syringe filter and this aliquot was used to measure the pH and determine total sugars by the phenol–sulfuric acid method [15].

### 2.3. pH Determination

The pH measurements were carried out on the analyzed juice samples (free of biomass and filtered through a 0.45 µm PTFE syringe filter). This allowed evaluating whether the experimental conditions tested were able to cause significant alterations in the pH values, since it is a variable to be considered in the stability of apple juices. All pH measurements were performed in triplicate using a sample volume of 4 mL with a Crison BASIC 20 pH-meter (Spain).

### 2.4. Determination of Biomass

The biomass concentration (g/L) was determined by turbidimetry at a wavelength of 600 nm using a double-beam V-650 spectrophotometer (Jasco, Madrid, Spain), but first a calibration curve was obtained by promoting the microbial growth in 100 mL of orange or apple juice, centrifuged, and filtered in the same conditions described above. For this purpose, juice matrices were spontaneously fermented over 7 days at 36 °C, in an orbital shaker, without agitation. After this time, solids containing biomass were separated by centrifugation at 4863 rcf, for 30 min at 4 °C. Afterwards, the biomass was washed twice with ultrapure water and the initial volume of the sample (100 mL) was returned with ultrapure water for subsequent turbidimetry measurements at 600 nm. To establish a correct relationship between the absorbance signal and the biomass concentration (g/L), three aliquots of the washed biomass suspension (1 mL) were dried in an oven at 105 °C for 48 h and the biomass dry weights were quantified [16]. Two biomass calibrations curves, in the range of 0.05–0.6 g biomass/L, were prepared to quantity the biomass concentration of all analyzed samples: one for orange juice (Absorbance = (2.605 ± 0.101) × biomass − (0.029 ± 0.014); r = 0.998) and other for apple juice (Absorbance = (1.935 ± 0.094) × biomass − (0.0497 ± 0.0247); r = 0.996). The limits of quantification (LOQ) obtained in both orange and apple juices were 0.129 and 0.133 g/L, respectively.

### 2.5. Determination of Total Soluble Sugars

The total content of soluble sugars was quantified in apple and orange juices, free of biomass, by following the method proposed by Dubois et al. [15]. Hence, 1 mL of juice, filtered through a 0.45 µm polytetrafluoroethylene (PTFE) syringe filter, was mixed with 1 mL of the phenol solution (50 g/L) and 5 mL of sulfuric acid (96% *w*/*w*). The mixture was gently stirred and allowed to cool for 10 min. Then, it was stirred again in a vortex and placed in a water bath at 30 °C for 20 min to promote the colorimetric reaction. Finally, the absorbance was quantified at 490 nm using a double-beam V-650 spectrophotometer (Jasco, Madrid, Spain). Phenol (99.5% *w*/*w*) and sulfuric acid (96% *w*/*w*) reagents were of analytical reagent grade and supplied both by Panreac (Barcelona, Spain). A calibration curve prepared with different standards of known concentration of glucose, in the range of 1–100 mg/L, was used to convert the absorbance signal in sugar concentration for all analyzed samples (Absorbance = (0.00964 ± 0.00198) × glucose + (0.0351 ± 0.0093); r = 0.999). The limit of quantification (LOQ) obtained for glucose determination was 0.296 mg/L.

### 2.6. Statistical Treatment of Data

The experimental results were statistically analyzed using the Design-Expert Version 13 software for Windows (Stat-Ease, Inc., Minneapolis, MN, USA), which provides mathematical equations that allowed predicting the effect of the biosurfactant extract in the two studied juice matrices. The equations fit a second-order polynomial based on Equation (1).

(1) $${y=\beta }_{0}+\beta_{1}x_{1}+\beta_{2}x_{2}+\beta_{3}x_{3}+\beta_{12}x_{1}x_{2}+\beta_{13}x_{1}x_{3}+\beta_{23}x_{2}x_{3}+\beta_{11}x_{1}^{2}+\beta_{22}x_{2}^{2}+\beta_{33}x_{3}^{2}$$

where y is the dependent variable, x represents the independent variables, and β corresponds to the regression coefficients calculated by the statistical program using the least squares method.

**Figure 1:**
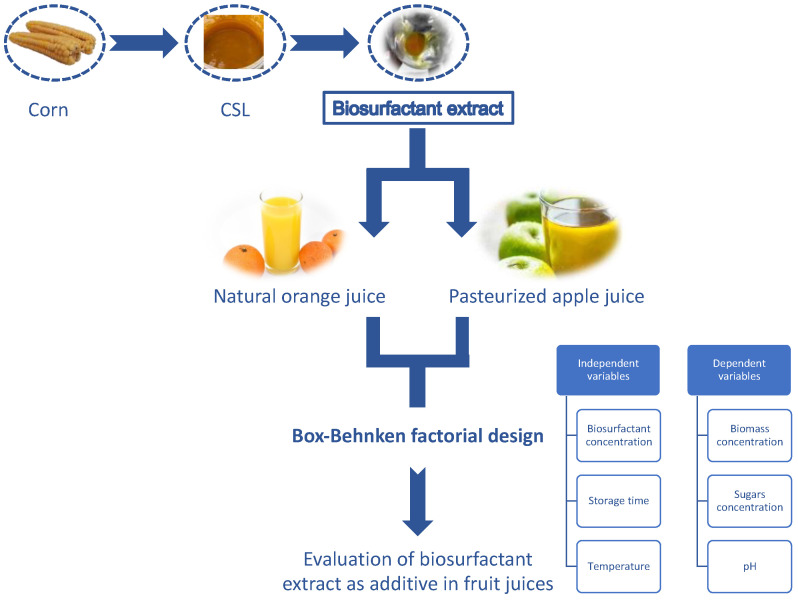
Proposed scheme to test the biosurfactant extract obtained from CSL as a food additive in fruit matrices.

Thus, the methodology of this study was performed following the scheme depicted in Figure 1.

## 3. Results and Discussion

### 3.1. Factorial Design to Evaluate the Stability of Fruit Juices

The stability of two different juice matrices (natural orange and pasteurized apple) was evaluated under different experimental conditions such as presence or absence of a CSL biosurfactant extract and different storage times and temperatures by conducting a Box–Behnken factorial design. The variables quantified experimentally in the different trials were the biomass production (y_1_), the total sugars concentration (y_2_), and pH (y_3_). The multifunctional biosurfactant extract included in the juice’s matrices contains lipopeptides composed of C16 and C18 fatty acids, such as palmitic, stearic, oleic, and linoleic acid as well as antioxidants [13]. It is amphoteric and possesses a critical micellar concentration (CMC) of 441 mg/L, reducing the surface tension of water by up to 39.5 mN/m. In addition, this biosurfactant extract has a strong antimicrobial activity against pathogenic bacteria such as *Staphylococcus aureus*, *Escherichia coli*, and *Pseudomonas aeruginosa* [17].

Table 3 shows the results obtained for the dependent variables studied (y_1_–y_3_) in the 15 experiments developed in the factorial design; Table 4 shows the coefficients obtained after the statistical treatment of data. Theses coefficients allowed us to obtain theoretical equations to predict the behavior of the dependent variables in the ranges established in Table 1 for the independent variables (x_1_–x_3_).

These mathematical equations were configured by considering only the coefficient for β_0_ and the significant regression coefficients (*p* < 0.05) for both orange juice (Equations (2)–(4)) and apple juice (Equations (5)–(7)) related to the independent variables:

y_1_ = 1.02 + 0.356 x_2_ − 0.715 x_3_^2^ (2)

y_2_ = 151.9 − 31.21 x_2_^2^ (3)

y_3_ = 3.48 − 0.0525 x_3_ + 0.150 x_2_^2^ + 0.100 x_3_^2^ (4)

y_1_ = 1.19 + 0.413 x_2_ − 0.817 x_3_^2^ (5)

y_2_ = 102.85 − 7.69x_1_ + 7.07x_2_ − 10.67x_12_ − 37.41x_2_^2^ (6)

y_3_ = 3.38 − 0.0413x_1_ − 0.0229x_1_^2^ + 0.0671x_2_^2^ + 0.0246x_3_^2^ (7)

From the *p*-values of the coefficients (Table 4) corresponding to the independent variables, it can be observed that the most significant variables on biomass concentration (y_1_) were the storage time (x_2_) and temperature (x_3_) in both juices (Equations (2) and (5)), whereas the sugar concentration and pH were affected in different ways by the studied independent variables. Hence, biosurfactant concentration (x_1_) and storage time (x_2_) were the more significant variables in apple juice when the sugar concentration (y_2_) was considered (Equation (6)), whereas in orange juice (Equation (3)) the only significant variable was the storage time (x_2_). Regarding pH, apple juice (Equation (7)) was affected by the three studied independent variables, the concentration of biosurfactant extract (x_1_) being the variable that provided the most significant. However, in orange juice the pH was not affected by the biosurfactant concentration, instead being mainly affected by temperature (x_3_) followed by the storage time (x_2_) (Equation (4)). It is important to remark that although the changes in pH were statistically affected by most of the studied independent variables, this supposes minimum changes in the experimental pH values always ranging between 3.35 and 3.48 in apple juice and between 3.40 and 3.77 in orange juice.

### 3.2. Effect of a Biosurfactant Extract in Pasteurized Apple Juice

**Figure 2:**
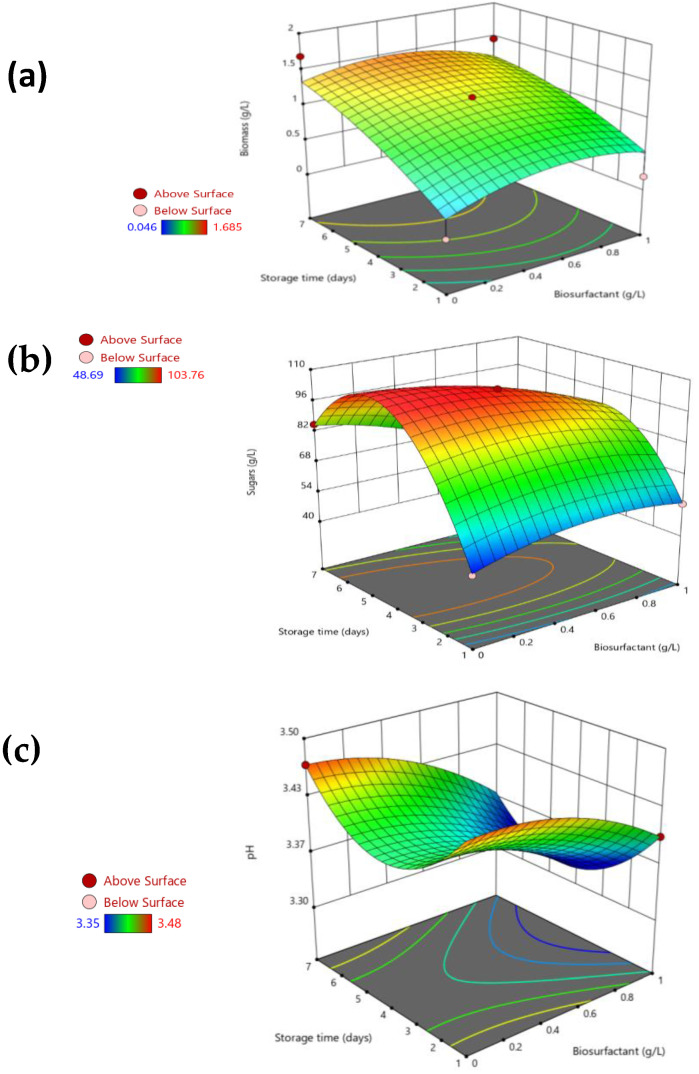
3D response–surface plots showing the variation of the three dependent variables ((**a**) biomass, (**b**) sugars, and (**c**) pH) in pasteurized apple juice with the biosurfactant concentration (g/L) and the storage time (days) at a fixed temperature of 20 °C.

Figure 2 shows the response-surface plots that depict the variation of the three studied dependent variables (y_1_–y_3_) against the concentration of biosurfactant extract and the storage time, for a fixed temperature of 20 °C, since temperature was the variable that showed the least significant effect on the studied system (Equations (5)–(7)). Figure 2a shows the effect of the biosurfactant extract on biomass production; a negligible effect of the biosurfactant concentration was observed, although after 4–5 days of storage a slight decrease in biomass at concentrations of biosurfactant higher than 0.5 g/L was observed in comparison with those apple juice matrices with lower biosurfactant extract concentrations. The maximum values of biomass of about 1.68 g/L were obtained at 7 days of storage in absence of biosurfactant extract, whereas in the presence of 1 g/L of biosurfactant the biomass production decreased until 1.46 g/L for the same incubation time and intermediate temperature (20 °C). Moreover, at intermediated conditions of temperature (20 °C), concentration of biosurfactant extract (0.5 g/L), and storage time (4 days), the biomass achieved was 1.18 g/L–1.21 g/L, showing a good agreement between the experimental and theoretical data (1.19 g/L). In comparison with those experiments carried out in the absence of biosurfactant extract, the reduction observed in biomass when the concentration of biosurfactant was increased above 0.5 g/L could be due to the antimicrobial capacity of the biosurfactant extract over these concentrations. Hence, López-Prieto et al. [17] found that the biosurfactant extract under evaluation inhibited the growth of *E. coli* by 75% at concentrations of 0.5 g/L and achieved 100% inhibition at a concentration of 1 g/L, among other pathogenic bacteria. In this sense, it is important to indicate that the most frequent microorganisms that can be found in spoiled apple juice are *Escherichia coli* O157:H7, *Salmonella Typhimurium*, and *Listeria monocytogenes* [18,19]. In addition, the fungicidal activity of the same biosurfactant extract from CSL against *A. brasiliensis,* which may affect foods, was also proven. Therefore, a 100% inhibition against *A. brasiliensis* at biosurfactant concentrations of 0.33 mg/mL at 4 °C was observed [20]. Following with the analysis of the results from the current work, it should be noted that the same trend was also observed at a temperature of 4 °C, although bacterial proliferation in this case was lower, as at this temperature the microbial growth is partially inhibited.

Regarding the concentration of total sugars, Figure 2b represents the variation of this parameter with the storage time and the biosurfactant concentration at 20 °C. In general, it was observed that total sugars increased up to 6 days of storage, reaching a content of around 99 g/L in the absence of biosurfactant extract. The increase of total sugars is due to the solubilization of simple sugars in the supernatant of apple juice, as apple juice is composed of pectins that release sugars to the aqueous phase by the hydrolytic action induced by the organic acids produced by microbial biomass. In the initial stage of the storage, sugars contained in the non-soluble pectin polymeric matrix of apple juice were partially removed by centrifugation. However, with the increase of microbial biomass, sugars were released from polymeric pectin and solubilized in the supernatant. Next, after 6 days of storage, the hydrolysis rate of sugars detected in the juice supernatant was lower than the degradation of sugars by the microbial biomass contained in the apple juice, and thus a decrease of sugars in the juice supernatant was observed. Comparing the data of experiments 6 and 8, included in Table 3, it was observed that in the absence of biosurfactant extract under an intermediate temperature (20 °C) and maximum storage time (7 days), the supernatant of apple juice contained 85.4 g/L of sugars, whereas under the same conditions of storage and temperature but in the presence of 1 g/L of biosurfactant the content of sugars was reduced by up to 49.0 g/L. From Figure 2b, it is also observed that concentrations of biosurfactant extract higher than 0.4 g/L promote a lower release of sugars in the supernatant of apple juice in comparison with experiments in the absence of or with lower concentrations of biosurfactants. Thus, at intermediate conditions of temperature (20 °C), storage time (4 days), and concentration of biosurfactant extract (0.5 g/L), as shown in Figure 2b, it is predicted that there would be 102.8 g/L of sugars in the apple juice supernatant under the same conditions, but without the addition of the biosurfactant extract (0 g/L, 20 °C and 4 days) the predicted concentration is 104.6 g/L. This fact can be related to the lower concentration of hydrolytic microbial biomass and organic acids in presence of high concentrations of biosurfactants. Hence, it can be speculated that the biosurfactant extract obtained from CSL preserves polymeric non-soluble sugars from hydrolysis, promoting the shelf-life of apple juice, which could reduce the glycemic index of the juice. Sandi et al. [21] evaluated the profile of reducing sugars in pasteurized yellow passion fruit juice at different heating temperatures (75–85 °C) and storage times for 120 days at room temperature (25 °C) or under refrigeration (5 °C), observing that reducing sugars (glucose and fructose) increased significantly over time. Moreover, the increase in the reducing sugar was higher in the juices pasteurized at higher temperatures and kept at room temperature. Other authors [22] also confirmed the significant increase of total sugars in different pasteurized fruit juices, including apple juice, after thirty days of storage time at refrigeration temperature but evaluating the sugar progressions after ten day intervals. Moreover, Wang et al. [23] evaluated the effects of storage temperature and time on the stability of concentrated apple juice. These authors observed that the increase in temperature (0 to 20 °C) and storage time (0, 15, 30, 60 to 90 days) caused a decrease in the total sugar content from 117 to 112 g/L or from 117 to 110 g/L when the storage time was extended up to 90 days or the incubation temperature increased from 0 to 20 °C, respectively, probably due to the rate of soluble sugar consumption by the microbial biomass being higher than the rate of hydrolysis of polymeric sugars.

In addition, Figure 2c shows the variation of pH with both the concentration of biosurfactant and storage time at intermediate temperature (20 °C) achieved in the current work. The addition of the biosurfactant extract to the pasteurized apple juice affected the pH values, causing a slight reduction when increasing the concentration of the biosurfactant extract, but the changes were minimal and the pH values ranged from 3.35 to 3.48, suggesting that the biosurfactant extract maintained the chemical properties and stability of apple juice regarding pH variations. These pH values are in good agreement with those previously reported in apple juice, which were increased from 3.91 to 4.13 when the storage time was extended from 0 to 90 days [22,23].

### 3.3. Effect of the Biosurfactant Extract in Natural Orange Juice

**Figure 3:**
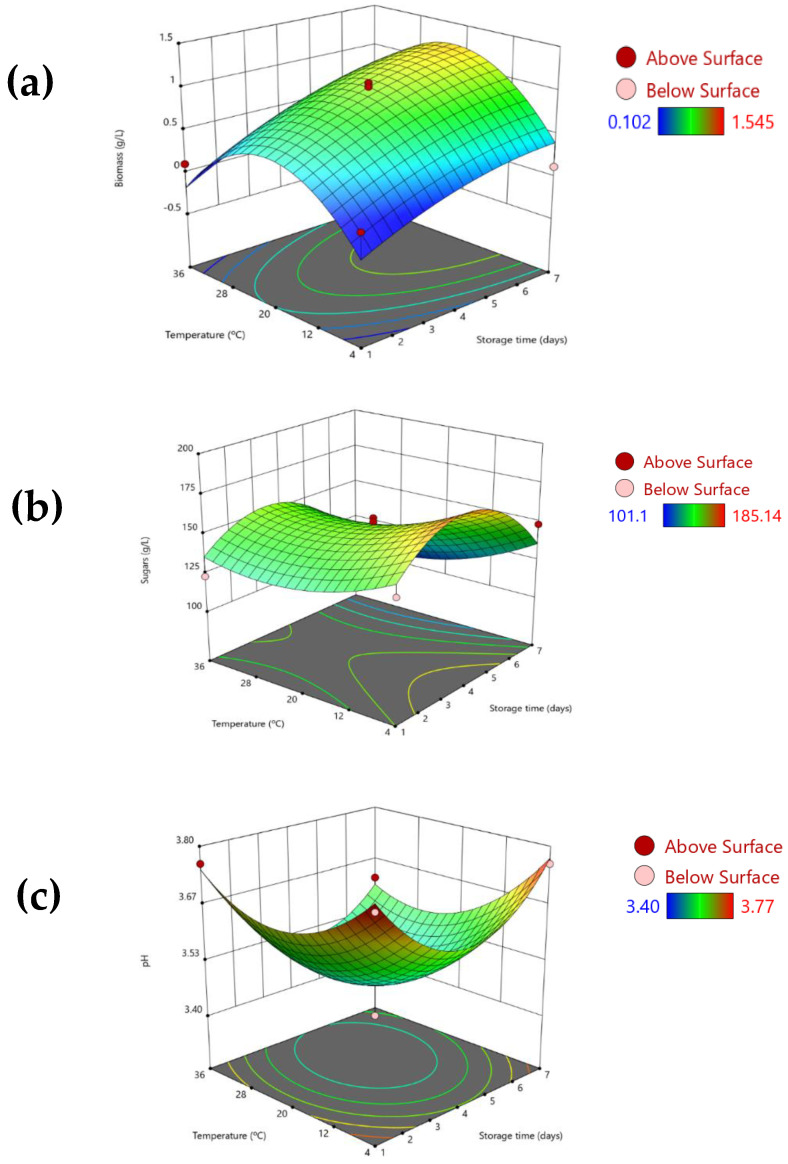
3D response–surface plots showing the variation of the three dependent variables ((**a**) biomass, (**b**) sugars, and (**c**) pH) in natural orange juice with the storage time (days) and the temperature (°C), fixing the concentration of the biosurfactant extract at 0.5 g/L.

From the independent variables assayed on orange juice, the concentration of biosurfactant extract was the least significant (Equations (2)–(4)), and thus Figure 3 includes the variation of the studied dependent variables (y_1_–y_3_) with temperature and storage time, fixing the concentration of biosurfactant at an intermediate value of 0.5 g/L.

Figure 3a shows the variation of biomass with temperature and storage time, showing that the biomass increased with a longer incubation time and intermediate temperature, achieving a value up to 1.28 g/L when the juice was stored over 7 days at 20 °C in the presence of 1 g/L of biosurfactant extract (experiment 8 of Table 3), the concentration of biomass being 1.54 g/L in the absence of biosurfactant extract (experiment 6 of Table 3). This value is close to the optimal biomass production zone achieved in apple juice (Figure 2a) and is consistent with the growth interval of the main microorganisms present in orange juice. As shown in Figure 3a, it was observed that biomass growth was lower at storage times shorter than 7 days and at extreme temperatures (4 °C and 37 °C). Cevallos-Cedeño and Velásquez-Murillo [24] found that lactic acid bacteria grows at temperatures ranging from 5 to 53 °C with an optimum growth range of 30 to 45 °C in orange juice; *Leuconostoc* lactic bacteria, whose growing interval is from 10 to 37 °C, the optimum was from 20 to 30 °C, and for the fungi *Ascomycetes* type, the optimum growth temperature ranges was from 20 to 30 °C [24]. Regarding the concentration of totals sugars in orange juice supernatant, the presence of the biosurfactant extract had a negligible effect (Equation (3)). Figure 3b shows the variation of sugar concentration in orange juice supernatant with temperature and storage time, fixing the biosurfactant concentration at 0.5 g/L. The profile of sugar concentration followed the same performance as that of apple juice regarding the storage time, observing an increase of sugars in supernatant up to 4 days of storage, but decreasing after this time. Thus, the minimum sugar content, between 101 g/L and 115 g/L, was detected in experiments 8, 6, and 4, developed at the maximum storage time under different temperatures (36 °C and 20 °C) and different concentrations of biosurfactant extract. When the temperature was fixed at 4 °C, the concentration of sugars was high (148 g/L), even for those experiments with longer storage times of 7 days (see Table 3, experiment 2), although the highest experimental concentration (185 g/L) was detected a reduced temperature (4 °C) and 4 days of storage time (see Table 3, experiment 9). However, in this case, no significant effect caused by the biosurfactant extract in the sugar release and their consumption along storage time was noticed, contrarily to the results described for apple juice. This might be related to the differences in microbial biomass growth in both juice matrices. Sandi et al. [21] also found a higher increase in glucose and fructose in juices pasteurized at higher temperatures and with storage at room temperature; thus, the pasteurization process can promote the hydrolysis and solubilization of sugars to juice supernatant. Additionally, Dhenge et al. [25] evaluated the quality of orange juice stabilized by two thermal (helical heat exchanger and ohmic heating) and non-thermal treatments (high-pressure processing), observing that a mild pasteurization process promoted the solubilization of the water-soluble compounds without leading to their degradation because of the mild temperatures and short time. Pham et al. [26] reported that reducing sugars in orange juice, including glucose and fructose, caused an increase in concentration during storage, as they can be generated from acid and soluble invertase-catalyzed sucrose hydrolysis [27,28]. They expected to observe a decrease in the glucose and fructose contents in the later phase of storage for low-pH samples (1.5 and 2.5) due to the complete inversion of sucrose in the early phase of storage; however, this change was not observed in this work. The authors postulated that a small degradation might not significantly change the sugar content because the contents of glucose and fructose in the orange juice were high [26].

The initial sample of natural orange juice studied yielded a pH of 3.52 and Figure 3c shows the variation of pH with temperature and storage time at intermediate concentrations of biosurfactant extract (0.5 g/L), being in the range of 3.40–3.77. It is important to indicate that the usual pH values on orange juice range from 3.30 to 4.19 depending on the orange variety used [29]. As shown in Figure 3c, a scarce decrease in pH values in orange juice was observed when incubated at intermediate times and temperatures (around 4 days and 20 °C), promoting the fermentation of the juice and the production of organic acid metabolites in this condition, which can produce variations in pH, although these variations can be considered negligible.

## 4. Conclusions

In this work, a multifunctional biosurfactant extract obtained from CSL was incorporated into two fruit juices (natural orange and pasteurized apple) to evaluate its influence on the stability and shelf-life of the juice matrices. A Box–Behnken factorial design was conducted to assess the role of the biosurfactant extract concentration on the biomass growth, sugar concentrations, and pH when the juices were stored between 1 and 7 days at different conservation temperatures (4–36 °C). The results obtained showed that while the presence of the biosurfactant extract was negligible for all dependent variables quantified in orange juice, it could be considered a significant variable in pasteurized apple juice regarding both sugar content and pH. Thus, it was observed that the hydrolysis rate of polymeric sugars in apple juice was reduced (85.4 to 49.0 g/L) in the presence of concentrations of biosurfactant extract higher than 0.4 g/L, in comparison with samples without or with low concentrations of biosurfactant extract. In contrast, pH changes in apple juice (from 3.35 to 3.48) were statistically affected by the presence of biosurfactant extract, although the changes in pH were negligible, suggesting that the presence of the biosurfactant extract did not modify its chemical properties and stability. Moreover, the biosurfactant extract almost did not affect the biomass growth in both studied fruit juices; however, a moderate decrease of biomass production in apple juice was observed 4–5 days after storage at biosurfactant concentrations higher than 0.5 g/L. This study is relevant because it is the first time that a natural biosurfactant extract has been included in a juice matrix, obtaining interesting results. It was observed that the biosurfactant extract produced a different effect regarding sugar hydrolysis and microbial consumption depending on the matrix. In apple juice, it reduced the presence of soluble sugars, whereas in orange juice this parameter was not affected. Based on this work, the biosurfactant extract from CSL could be considered a potential additive in fruit juices, particularly in apple juice, to promote its microbial stability and reduce the release of soluble sugar content during storage. Nevertheless, to be able to carry out a complete inclusion of these biocompounds in food matrices, additional food toxicology studies are necessary and more studies on different juice matrices are also recommended.

## Figures and Tables

**Table 1:** Independent and dependent variables evaluated through the Box–Behnken factorial design.

**Independent Variables**			
	**Units**	**Range**	
Biosurfactant concentration	g/L	0–1	
Storage time	days	1–7	
Temperature	°C	4–36	
**Coded Independent Variables**			
	**Nomenclature**	**Equation**	**Range**
Biosurfactant concentration	x_1_	(x_1_ − 0.5)/0.5	(−1, 1)
Storage time	x_2_	(x_2_ − 4)/3	(−1, 1)
Temperature	x_3_	(x_3_ − 20)/16	(−1, 1)
**Dependent Variables**			
	**Nomenclature**	**Units**	
Biomass concentration	y_1_	g/L	
Sugars concentration	y_2_	g/L	
pH	y_3_	---	

**Table 2:** Experiments and independent variables corresponding to the Box–Behnken factorial design. Biosurfactant concentration (x_1_), storage time (x_2_), and temperature (x_3_).

	Non Coded Independent Variables			Coded Independent Variables		
Experiment	x_1_	x_2_	x_3_	x_1_	x_2_	x_3_
1	0.5	1	4	0	−1	−1
2	0.5	7	4	0	1	−1
3	0.5	1	36	0	−1	1
4	0.5	7	36	0	1	1
5	0	1	20	−1	−1	0
6	0	7	20	−1	1	0
7	1	1	20	1	−1	0
8	1	7	20	1	1	0
9	0	4	4	−1	0	−1
10	0	4	36	−1	0	1
11	1	4	4	1	0	−1
12	1	4	36	1	0	1
13	0.5	4	20	0	0	0
14	0.5	4	20	0	0	0
15	0.5	4	20	0	0	0

**Table 3:** Experimental results corresponding to the dependent variables quantified in the Box–Behnken factorial design for both apple juice and orange juice. Biomass (y_1_) is expressed in g/L; total sugars (y_2_) are expressed in g/L and pH (y_3_).

	y_1_	y_2_	y_3_	y_1_	y_2_	y_3_
Expt	Apple Juice			Orange Juice		
1	0.107 ± 0.005	57.1 ± 0.2	3.47 ± 0.03	0.107 ± 0.004	144 ± 6	3.77 ± 0.01
2	0.151 ± 0.001	56.6 ± 1.0	3.48 ± 0.01	0.120 ± 0.001	148 ± 5	3.76 ± 0.01
3	0.191 ± 0.009	52.7 ± 0.5	3.48 ± 0.01	0.102 ± 0.002	123 ± 6	3.76 ± 0.01
4	0.566 ± 0.001	78.8 ± 3.7	3.47 ± 0.03	0.351 ± 0.011	115 ± 5	3.62 ± 0.02
5	0.075 ± 0.003	48.7 ± 0.2	3.48 ± 0.01	0.110 ± 0.003	143 ± 2	3.75 ± 0.01
6	1.68 ± 0.01	85.4 ± 1.1	3.47 ± 0.02	1.54 ± 0.02	101 ± 4	3.64 ± 0.01
7	0.188 ± 0.009	54.9 ± 0.1	3.40 ± 0.02	0.128 ± 0.003	146 ± 6	3.67 ± 0.01
8	1.46 ± 0.05	49.0 ± 0.8	3.36 ± 0.04	1.28 ± 0.01	102 ± 1	3.61 ± 0.01
9	0.046 ± 0.001	101 ± 1	3.43 ± 0.03	0.108 ± 0.001	185 ± 6	3.70 ± 0.01
10	0.130 ± 0.006	99.9 ± 0.1	3.41 ± 0.02	0.241 ± 0.009	135 ± 4	3.53 ± 0.01
11	0.165 ± 0.007	92.4 ± 1.0	3.35 ± 0.01	0.122 ± 0.005	165 ± 3	3.67 ± 0.01
12	0.28 ± 0.002	77.5 ± 3.6	3.35 ± 0.01	0.275 ± 0.005	180 ± 1	3.57 ± 0.02
13	1.18 ± 0.01	101 ± 3	3.38 ± 0.01	1.07 ± 0.01	145 ± 1	3.40 ± 0.01
14	1.21 ± 0.02	104 ± 4	3.39 ± 0.01	1.03 ± 0.01	157 ± 0	3.51 ± 0.02
15	1.18 ± 0.01	104 ± 2	3.38 ± 0.01	0.967 ± 0.005	154 ± 0	3.52 ± 0.02

**Table 4:** Regression coefficients (β) and their statistical significance (*p-*value) for the dependent variables biomass (y_1_); total sugars (y_2_); and pH (y_3_) for both apple juice and orange juice.

Apple Juice						
Coeff.	y_1_	*p*-Values	y_2_	*p*-Values	y_3_	*p*-Values
β_0_	1.19	0.1309	102.8	0.0013 *	3.38	0.0010 *
β_1_	0.0201	0.8917	−7.69	0.0111 *	−0.0413	0.0002 *
β_2_	0.413	0.0323 *	7.07	0.0154 *	−0.0063	0.2064
β_3_	0.0875	0.5607	0.196	0.9241	−0.0025	0.5867
β_1_β_2_	−0.0840	0.6900	−10.67	0.0120 *	−0.0075	0.2729
β_1_β_3_	0.0082	0.9685	−3.39	0.2764	0.0050	0.4490
β_2_β_3_	0.0827	0.6943	6.66	0.0615	−0.0050	0.4490
β_1_^2^	−0.2179	0.3402	−5.93	0.0951	−0.0229	0.0153 *
β_2_^2^	−0.1199	0.5871	−37.41	< 0.0001 *	0.0671	0.0001 *
β_3_^2^	−0.8167	0.0109 *	−4.13	0.2116	0.0246	0.0117 *
**Orange Juice**						
β_0_	1.02	0.171	151.9	0.0626	3.48	0.0229 *
β_1_	−0.0252	0.855	3.53	0.511	−0.0125	0.511
β_2_	0.356	0.0424 *	−11.19	0.0750	−0.0400	0.0732
β_3_	0.0640	0.647	−11.29	0.0731	−0.0525	0.0312 *
β_1_β_2_	−0.0715	0.716	−0.517	0.944	0.0125	0.638
β_1_β_3_	0.00500	0.979	16.33	0.0685	0.0175	0.515
β_2_β_3_	0.0590	0.764	−3.11	0.678	−0.0325	0.251
β_1_^2^	−0.120	0.561	2.20	0.777	0.0400	0.181
β_2_^2^	−0.137	0.511	−31.21	0.0081 *	0.150	0.0022 *
β_3_^2^	−0.715	0.0140 *	11.93	0.165	0.100	0.0119 *

* *p* < 0.05 indicates significant variables for confidence interval of 95%.

## Data Availability

All related data and methods are presented in this paper. Additional inquiries should be addressed to the corresponding author.

## References

[1] Sarubbo L.A., Maria da Gloria C.S., Durval I.J.B., Bezerra K.G.O., Ribeiro B.G., Silva I.A., Twigg M.S., Banat I.M. (2022). Biosurfactants: Production, Properties, Applications, Trends, and General Perspectives. Biochem. Eng. J..

[2] Gudiña E.J., Rocha V., Teixeira J.A., Rodrigues L.R. (2010). Antimicrobial and Antiadhesive Properties of a Biosurfactant Isolated from *Lactobacillus Paracasei Ssp. Paracasei* A20. Lett. Appl. Microbiol..

[3] Vecino X., Rodríguez-López L., Ferreira D., Cruz J.M., Moldes A.B., Rodrigues L.R. (2018). Bioactivity of Glycolipopeptide Cell-Bound Biosurfactants against Skin Pathogens. Int. J. Biol. Macromol..

[4] Durval I.J.B., Ribeiro B.G., Aguiar J.S., Sarubbo L.A., Rufino R.D., Converti A. (2021). Application of a Biosurfactant Produced by *Bacillus Cereus* Ucp 1615 from Waste Frying Oil as an Emulsifier in a Cookie Formulation. Fermentation.

[5] Ghazala I., Charfeddine S., Charfeddine M., Gargouri R., Semia B., Chaabouni E. (2022). Antimicrobial and Antioxidant Activities of *Bacillus Mojavensis* I4 Lipopeptides and Their Potential Application against the Potato Dry Rot Causative Fusarium Solani. Arch. Microbiol..

[6] Mouafo H.T., Sokamte A.T., Mbawala A., Ndjouenkeu R., Devappa S. (2022). Biosurfactants from Lactic Acid Bacteria: A Critical Review on Production, Extraction, Structural Characterization and Food Application. Food Biosci..

[7] Hoffmann M., Mück D., Grossmann L., Greiner L., Klausmann P., Henkel M., Lilge L., Weiss J., Hausmann R. (2021). Surfactin from *Bacillus Subtilis* Displays Promising Characteristics as O/W-Emulsifier for Food Formulations. Colloids Surf. B Biointerfaces.

[8] Ribeiro B.G., Campos Guerra J.M., Sarubbo L.A. (2022). Production of a Biosurfactant from *S. Cerevisiae* and Its Application in Salad Dressing. Biocatal. Agric. Biotechnol..

[9] López-Prieto A., Rodríguez-López L., Rincón-Fontán M., Moldes A.B., Cruz J.M. (2019). Effect of Biosurfactant Extract Obtained from the Corn-Milling Industry on Probiotic Bacteria in Drinkable Yogurt. J. Sci. Food Agric..

[10] Ravindran A., Kiran G.S., Selvin J. (2022). Revealing the Effect of Lipopeptide on Improving the Probiotics Characteristics: Flavor and Texture Enhancer in the Formulated Yogurt. Food Chem..

[11] Martínez-Arcos A., López-Prieto A., Rodríguez-López L., Pérez-Cid B., Vecino X., Moldes A.B., Manuel C.J. (2021). Evaluation of Morphological Changes in Grapes Coated with a Biosurfactant Extract Obtained from Corn Steep Liquor. Appl. Sci..

[12] Scalzini G., López-Prieto A., Paissoni M.A., Englezos V., Giacosa S., Rolle L., Gerbi V., Segade S.R., Cid B.P., Moldes A.B. (2020). Can a Corn-Derived Biosurfactant Improve Colour Traits of Wine? First Insight on Its Application during Winegrape Skin Maceration versus Oenological Tannins. Foods.

[13] Rodríguez-López L., Vecino X., Barbosa-Pereira L., Moldes A.B., Cruz J.M. (2016). A Multifunctional Extract from Corn Steep Liquor: Antioxidant and Surfactant Activities. Food Funct..

[14] Box G.E., Hunter J.S., Hunter W.G. (2005). Statistics for Experimenters: Design, Innovation and Discovery.

[15] Dubois M., Gilles K.A., Hamilton J.K., Rebers P.A., Smith F. (1956). Colorimetric Method for Determination of Sugars and Related Substances. Anal. Chem..

[16] Bustos G., Arcosa U., Vecino X., Cruz J.M., Moldes A.B. (2018). Recycled *Lactobacillus pentosus* biomass can regenerate biosurfactants after various fermentative and extractive cycles. Biochem. Eng. J..

[17] López-Prieto A., Vecino X., Rodríguez-López L., Moldes A.B., Cruz J.M. (2019). A Multifunctional Biosurfactant Extract Obtained from Corn Steep Water as Bactericide for Agrifood Industry. Foods.

[18] Mendes-Oliveira G., Deering A.J., San Martin-Gonzalez M.F., Campanella O.H. (2020). Microwave Pasteurization of Apple Juice: Modeling the Inactivation of *Escherichia coli* O157:H7 and Salmonella Typhimurium at 80–90 °C. Food Microbiol..

[19] Kim S.S., Park J., Park H., Hong H., Kang D.H. (2020). Combined Ohmic Heating and Krypton-Chlorine Excilamp Treatment for the Inactivation of *Listeria Monocytogenes*, *Salmonella Typhimurium*, and *Escherichia Coli* O157:H7 in Apple Juice. J. Food Saf..

[20] López-Prieto A., Vecino X., Rodríguez-López L., Moldes A.B., Cruz J.M. (2021). Fungistatic and Fungicidal Capacity of a Biosurfactant Extract Obtained from Corn Steep Water. Foods.

[21] Sandi D., Paes Chaves J.B., Gomes De Sousa A.C., Maia Parreiras J.F., Coelho Da Silva M.T., Lessa Constant P.B. (2004). Hunter Color Dimensions, Sugar Content and Volatile Compounds in Pasteurized Yellow Passion Fruit Juice (Passiflora Edulis Var. Flavicarpa) during Storage. Braz. Arch. Biol. Technol..

[22] Rehman M.A., Khan M.R., Sharif M.K., Ahmad S., Shah F.-U.-H. (2014). Study on the Storage Stability of Fruit Juice Concentrates. Pakistan J. Food Sci..

[23] Wang H., Yuan J., Chen L., Ban Z., Zheng Y., Jiang Y., Jiang Y., Li X. (2022). Effects of Fruit Storage Temperature and Time on Cloud Stability of Not from Concentrated Apple Juice. Foods.

[24] Cevallos-Cedeño R.E., Velásquez-Murillo L.D. (2007). Engineering Thesis of Comparison of Temperature-Pasteurization Retention Time and Its Effect on Vitamin C Concentration in Orange Juice.

[25] Dhenge R., Langialonga P., Alinovi M., Lolli V., Aldini A., Rinaldi M. (2022). Evaluation of Quality Parameters of Orange Juice Stabilized by Two Thermal Treatments (Helical Heat Exchanger and Ohmic Heating) and Non-Thermal (High-Pressure Processing). Food Control.

[26] Pham H.T.T., Kityo P., Buvé C., Hendrickx M.E., Van Loey A.M. (2020). Influence of Ph and Composition on Nonenzymatic Browning of Shelf-Stable Orange Juice during Storage. J. Agric. Food Chem..

[27] Wibowo S., Grauwet T., Santiago J.S., Tomic J., Vervoort L., Hendrickx M., Van Loey A. (2015). Quality Changes of Pasteurised Orange Juice during Storage: A Kinetic Study of Specific Parameters and Their Relation to Colour Instability Scheling. Food Chem..

[28] Li M., Liu Q., Zhang W., Zhang L., Zhou L., Cai S., Hu X., Yi J. (2021). Evaluation of Quality Changes of Differently Formulated Cloudy Mixed Juices during Refrigerated Storage after High Pressure Processing. Curr. Res. Food Sci..

[29] FDA/Center for Food and Drug Administration (2008). Approximate PH of Foods and Food Products.

